# Anthocyanins and Their C_6_-C_3_-C_6_ Metabolites in Humans and Animals

**DOI:** 10.3390/molecules24224024

**Published:** 2019-11-07

**Authors:** Wilhelmina Kalt

**Affiliations:** Agriculture & Agri-Food Canada (Retired). 212 Foley Road, RR#3 Centreville, NS B0P 1J0, Canada; wilhelmina.kalt@icloud.com; Tel.: +1-902-300-9042

**Keywords:** bioavailability, enterohepatic, flavonoid, phase 2 metabolism

## Abstract

Research on the bioavailability of anthocyanins has focused, historically, on the non-flavonoid (C_6_-C_n_) products that arise from anthocyanins in vivo. However, this review focuses on the products of anthocyanins that still possess the flavonoid structure (C_6_-C_3_-C_6_). Described herein are aspects of the in vivo pool of C_6_-C_3_-C_6_ anthocyanin-derived intermediates. Properties related to molecular size, shape, and polarity conveyed by six major anthocyanidin structures are discussed. The presence of a glycoside or not, and a variety of possible phase 2 conjugates, gives rise to a chemically diverse pool of C_6_-C_3_-C_6_ intermediates. Chemical properties influence the in vivo stability of anthocyanin-derived products, as well as their suitability as a substrate for xenobiotic conjugation and transport, and their association with the biomatrix. The flavonoid structure is associated with bioactivity and the particular properties of these C_6_-C_3_-C_6_ products of anthocyanins determines their deposition in the body, which may influence in vivo processes and ultimately health outcomes.

## 1. Introduction

**Figure 1:**
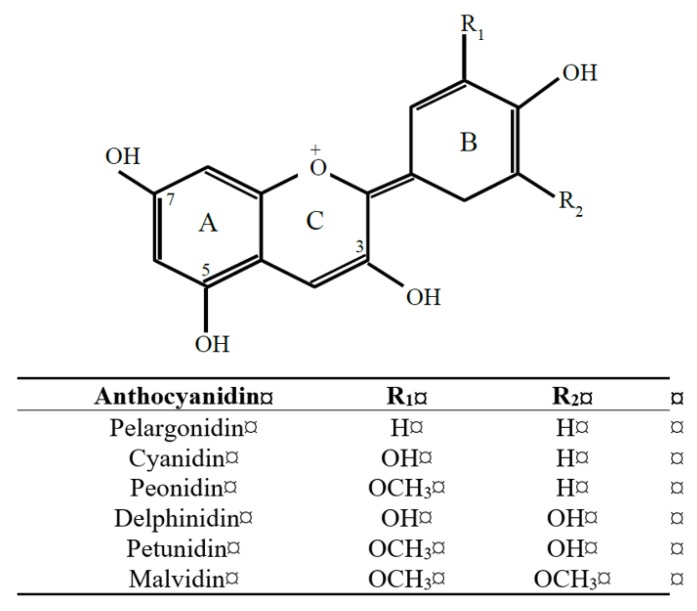
Generic structure of the six major anthocyanidins found in plant foods. Sugar conjugates of anthocyanidins (i.e., anthocyanins) occur most commonly via an *O*-linkage at carbon-3 and/or carbon-5. Phase 2 methylation, glucuronidation, sulfation is possible at all hydroxyl groups, giving rise to multiple positional isomers.

Anthocyanidin pigments (Figure 1) are widely distributed in fruit, especially berries, and can be the most abundant flavonoid in deeply pigmented berry crops like blueberries. Like other flavonoids [1,2], anthocyanin pigments continue to be investigated for their human health benefits. The collective evidence suggests that anthocyanin intake is associated with anti-oxidation, anti-inflammation [3], and vaso-modulation benefits by acting as biochemical effectors via multiple mechanisms. Mechanisms of anthocyanin action are being elucidated using in vitro, in vivo, and clinical research approaches [4], which is supported by evidence from epidemiological (observational) analyses that specifically examine the contribution of anthocyanin intake to health outcomes and disease risk.

Greater anthocyanin intake is associated with a decreased risk of all-cause mortality [5] which can be mainly accounted for by a reduced cardiovascular mortality risk [6]. There are several meta-analyses that associate greater anthocyanin intake with reduced cardiovascular disease risk [6,7,8,9,10] and with improved markers of cardiovascular health [11,12,13]. Greater anthocyanin intake is also associated with a reduced risk of type 2 diabetes [14] and better weight maintenance [15]. Beyond cardiovascular and metabolic function, anthocyanin intake is also associated with a delayed decline in cognition during aging [16].

In spite of a plethora of evidence for the beneficial effects of food forms of anthocyanin pigments (i.e., ‘parent anthocyanins,’ also called anthocyanidin glycosides) based on current bioavailability research, it is agreed that the concentration of parent anthocyanins in vivo would be too low to exert effects comparable to those observed in vitro [17]. It is also agreed that the absorption, digestion, metabolism and excretion (ADME) of anthocyanins is dynamic and complex. When anthocyanin ADME was investigated in a human study using ^13^C-labelled cyanidin-3-glucoside (C3g), rapid and complete anthocyanin breakdown was demonstrated when a significant proportion of the ^13^C -label was recovered as ^13^CO_2_ in breath. However 56% of the ^13^C label was still in the body 48 h after intake [18].

Factors that affect the dynamic retention of anthocyanins in the body are the focus of this review. The in vivo properties of anthocyanins, their interactions with abundant biomolecules, and the impact of human metabolism on them will be discussed. While the non-flavonoid products (i.e., C_6_-C_n_) of anthocyanins have been very well-studied [19], this review focuses on the C_6_-C_3_-C_6_-based products, which are worthy of attention, owing to their well-documented bioactivities [1,2].

## 2. Anthocyanin Properties in Vivo

### 2.1. Water Activity and Anthocyanin Stability

Whereas parent anthocyanins in aqueous conditions are unstable at neutral pH [20], they appear to be significantly stabilized in the biomolecular environment of the body [21,22,23], which relates at least in part to reduced hydration of flavylium ionic forms. For example, when pelargonidin glycoside’s stability was tested at different hydration states (using glycerol, at pH 3.4) greater water activity was associated with greater degradation with a 20-fold range in room temperature half-lives (56–934 days) [24]. When the relative in vitro stabilities of the three glucosides of cyanidin, delphinidin, pelargonidin and their aglycones were compared at neutral pH, B-ring structures with greater hydroxylation were associated with decreased stability [20]. 

The association of malvidin-3-glucoside (Mal3g) with anionic SDS micelles provided substantial stability and protected against pH-related bleaching [25]. The effect on stability was attributed to modified hydration and deprotonation of the Mal3glu molecule [25]. In another study using circular dichroism, anthocyanin stability was improved by association with anionic SDS micelles, with evidence of intermolecular self-association [26]. Improved stability of anthocyanins in lipophilic and amphipathic micellar environments has relevance to their behavior in vivo in bile and tissues. Improved flavonoid stability by emulsification has been reported [27] and appears to be important for anthocyanins as well. 

### 2.2. Membrane Solubility of Anthocyanins

**Figure 2:**
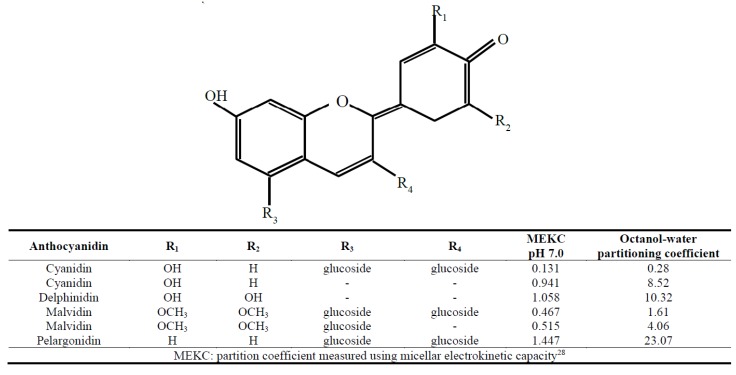
Partition coefficients of selected anthocyanidins and anthocyanins based on micellar electrokinetic methods and octanol-water partitioning [28].

Hydrophobicity. i.e., reduced polarity, is a key characteristic affecting the distribution of solutes between the lipophilic and hydrophilic environments of the cell. The behavior of solutes in the membrane can be modelled in vitro using micelles and liposome bilayers to provide a truer estimate of membrane solubility compared to the typical octanol-water partitioning, by taking into account the presence of membrane components. Using such methods, polarity was estimated using micellar electrokinetic chromatography (MEKC) and compared to octanol-water partitioning for a series of anthocyanin aglycones and glycosides in their uncharged forms at neutral pH (Figure 2) [28]. For both methods, cyanidin 3,5-diglucoside was the least membrane soluble, and substantially less so that cyanidin aglycone. Based on MEKC, pelargonidin 3,5-diglucoside showed the greatest capacity for partitioning into the membrane, similarly to delphinidin aglycone. Whereas octanol-water partitioning showed a strong effect of glycosylation, MEKC illustrated the more complex membrane partitioning behaviour of anthocyanidins, which is influenced by both their glycosylation and B-ring hydroxylation (Figure 2) [28].

Typically, deglycosylation of anthocyanins, which is rapid and extensive in vivo, produces an anthocyanidin (i.e., aglycone) which has significantly reduced molecular polarity and greater membrane solubility [29]. The reduced polarity of the small and less-polar molecule enables the paracellular transfer of solutes into cells, independent of membrane transporters, and therefore, provides a means to by-pass carrier-mediated transport.

### 2.3. Anthocyanin Association with Human Serum Albumin

The intense staining properties of anthocyanins are evidence of their propensity to adsorb to biomolecules, such as protein and polysaccharide [30]. Flavonoid adsorption to protein [31] is particularly relevant in relation to human serum albumin, which can both circulate and act as a depot for compounds. Anthocyanins interact with human serum albumin via hydrophobic and electrostatic interactions, which are favored by hydroxyl groups but not by methyl groups [32]. At pH 7.4, electrostatic interactions predominate, but at the acidic pH of the stomach, associations are due to hydrophobic effects [32]. Binding to human serum albumin was more favorable for anthocyanins than for anthocyanidins [32]. It has been suggested that the recovery of anthocyanins from plasma may be negatively affected by their binding to plasma proteins [33]. Anthocyanins are also reported to be associated with unidentified proteins in the stomach [22]. 

### 2.4. Anthocyanins in the Intestinal Tract

A mucus layer lines the gastrointestinal tract (GIT) which permits nutrient uptake and excludes bacteria and other toxic compounds. Evidence of a close physical association with mucin was suggested when almost 80% of anthocyanins administered could still be recovered from the small intestine of mice 2 h after gastric administration [22]. It is possible that a greater proportion of parent anthocyanin could survive first-pass metabolism when delivered in a large dose, if the capacity for first-pass metabolism becomes saturated. 

All that notwithstanding, there is evidence that anthocyanin uptake into enterocytes could be impeded by an anthocyanin association with mucin. Evidence for mucin’s effects include a study showing that when a large blackberry dose preceded a small dose, C3g absorption from the small dose was reduced compared to when the first dose was small [34]. Further evidence is that both total and parent anthocyanin content declined in human urine during 28 d of daily blueberry juice intake (*n* = 18) and even a 7 d wash-out did not change this declining trend [35]. Additionally, anthocyanin tissue bioavailability was markedly reduced in studies where a high, compared to a low, anthocyanin dose was used [36]. No anthocyanin was detected in brain, where it was expected, in a study that used high anthocyanin doses in food (500–600 mg/day) [37]. In a long-term human study, the urinary content of parent anthocyanins was greater when a daily blueberry juice dose was taken in 250 mL in the morning, rather than in three 83 mL doses over a 12 h period. This suggested that greater intestinal coverage improved the capacity for anthocyanin absorption [35].

In support of this notion, it has also been suggested that cationic anthocyanins bind to intestinal mucus [27]. But when anthocyanins are incorporated in mixed micelles with bile salts thereby changing their surface charge, anthocyanins can pass more readily through mucin due to modified solubility behavior [27,38]. It has also been hypothesized that anthocyanin stability is improved by binding to intestinal mucus, secretions, and food residues [21,22].

Closely associated with the mucin barrier is the gut-associated lymphoid tissue which conducts immune function for the host, in relation to gut and external microbiota [39]. Achieving translocation through the mucin layer and the enterocyte, makes anthocyanins available for association with capillaries and/or lymph vessels. Whereas hydrophilic solutes are mainly transported via plasma, hydrophobic solutes, including lipids, are transported in the lymph. It is interesting to consider how anthocyanin’s association with lymph could modulate immune function between the host and its gut microbiome. Phase 2 conjugates of quercetin were found in lymph, although quercetin aglycone was not [40].

With respect to food interactions in the GIT, when strawberries were eaten with cream, the T_max_ of pelargonidin products was delayed compared to without cream, but recovery was not affected [41]. It is possible that the presence of cream and fat delayed gastric emptying and the absorption of anthocyanins from the small and large intestine. A similar result was obtained with both quercetin glycoside and aglycone, whose absorptions were increased when delivered with high fat [42]. 

## 3. Uptake of Parent Anthocyanins on the Bilitranslocase

Intact parent anthocyanins can enter circulation directly and rapidly via the gastric bilitranslocase [43]. The bilitranslocase is an organic ionic membrane carrier whose best-characterized substrates are bilirubin, nicotinic acid, and sulfobromophtalein [43]. Whereas other flavonoids are not effective ligands for the bilitranslocase [44], anthocyanins meet the structural specifications, thereby providing a means for them to be absorbed directly without deglycosylation [43,45]. The structural interaction of anthocyanin on the bilitranslocase occurs preferentially via the glycoside moiety, thereby making anthocyanins better substrates than anthocyanidins [43]. 

Anthocyanins are transported across the bilitranslocase and into circulation in significant amounts. When glycosides of cyanidin, delphinidin, and malvidin contained in bilberries were administered to the stomach, absorption ranged between about 20% and 40%, with the greatest uptake being for delphinidins [34], noting that “absorption” could be due to uptake or loss. Anthocyanin uptake by the gastric bilitranslocase has been reported to correlate well with total intestinal uptake, suggesting that it is an important route for anthocyanin uptake [43]. Isoforms of the bilitranslocase are located at multiple sites, including the epithelia of the gastric cavity, jejunum, liver, kidney, and the vascular endothelium [43,44].

## 4. Parent Anthocyanin Conversion into C_6_-C_3_-C_6_ Products in Vivo

Anthocyanins, like other flavonoids, are subject to extensive first-pass metabolism involving intestinal and hepatic metabolic machinery, including conjugation enzymes and membrane transporters [27,44,46]. In humans, the profile of C_6_-C_3_-C_6_ conjugates of anthocyanins reflects the collective impact of substrate and location specificity in xenobiotic machinery, its phenotypic variation, and to a limited extent, microbial activity, plus the influences of the in vivo matrix. 

### 4.1. Deglycosylation

Anthocyanins occur in plants entirely as glycosidic forms, where they are localized in the cell’s large acidic vacuole. After ingestion by animals, anthocyanins are deglycosylated thereby releasing glycoside group(s) and anthocyanidins; i.e., aglycones. For many flavonoid glycosides, glycoside hydrolysis is necessary for aglycone absorption to occur [45].

Anthocyanidin glycoside hydrolysis can occur in the mouth via β-glycosidase activity, which is mainly of bacterial origin [47]. Anthocyanins in the small intestine can enter the enterocyte without deglycosylation via the sodium-coupled glucose transporter. And once inside the enterocyte, they can be deglycosylated via the cytosolic β-glycosidase. Otherwise anthocyanins can enter the enterocyte with glycoside hydrolysis on the lactase phlorizin hydrolase. Both the sodium-coupled glucose transporter and the lactase phlorizin hydrolase are localized on the apical side of the enterocyte [48]. 

Extensive deglycosylation of ^13^C-labelled C3g in humans was apparent, because none of the 24 ^13^C-labelled metabolites detected, were glycosylated [18]. However, it has been suggested that human β-glucosidase may be insufficient to totally deglycosylate anthocyanins due to very high phenotypic variation [49]. The intestinal β-glucosidase probably makes a greater contribution to total anthocyanin deglycosylation than does the liver’s β-glycosidase activity [48]. 

Food anthocyanins with less common glycosides, including acylated forms, can be resistant to human β-glycosidase activities and are more readily hydrolyzed by various bacterial β-glycosidases in the colon. Thus, the pharmacokinetics of mono-glycosidic anthocyanin forms can be distinct from anthocyanins with larger and more complex *O*-glycoside substitutions [50,51,52]. 

### 4.2. First-Pass Metabolism

Despite rapid and extensive deglycosylation, very few studies detect simple anthocyanidins (i.e., aglycone forms) in plasma and urine [53,54,55,56] which is consistent with the diminished aqueous solubility and stability of anthocyanidins. Anthocyanidins are substrates for phase 2 conjugation, involving glucuronidation via uridine 5′-diphosphate-glucuronosyltransferases, methylation via catechol-O-methyl transferase, and sulfation via sulfotransferases. Indeed anthocyanidins are strongly preferred over anthocyanins, as substrates for conjugation [45]. Typically, the most abundant phase 2 conjugates are methylated and glucuronidated anthocyanidins and anthocyanins [18,55,57,58,59,60,61]. Flavonoid glucuronidation occurs via uridine 5′-diphosphate-glucuronosyltransferase, which occurs in various isoforms at many different sites in the body [62,63]. Although phase 1 hydroxylation via cytochrome oxidases can occur, it is probably not a major pathway for flavonoids [45,62]. Notably, hydroxylation creates new reactive sites for phase 2 conjugation. Specificity in phase 2 conjugation enzymes and membrane carriers and efflux transporters, particularly those in the ATP-binding cassette transporter group, determine the site-specific in vivo pool of C_6_-C_3_-C_6_ metabolites derived from anthocyanins. 

## 5. Enterohepatic Circulation 

During enterohepatic circulation (EHC), solutes are selectively absorbed from the upper GIT and moved via the portal vein to the liver, where xenobiotic conjugation can occur. Solutes and their xenobiotic conjugates are incorporated into bile. Bile is released sporadically into the upper small intestine (ileum) to aid in food particle and fat absorption**.** After their release into bile, solutes including phase 2 conjugates, migrate down the GIT, where they can be deconjugated by human or bacterial enzymes. To remain in EHC, products are absorbed from the small and large intestine and returned to the liver via portal flow. To exit EHC, solutes and their metabolites are excreted in urine and feces or are deposited into tissue reservoirs [64]. EHC is manifested by a delayed clearance of a solute [64]. The most definitive and informative means to investigate EHC is with surgical cannulation models, which are possible in rodents but not in humans.

### 5.1. Factors Affecting EHC Uptake

Chemical factors that influence the capacity for a compound to be taken up into EHC include molecular weight (MW), structure, and polarity, with MW being the most consistent factor [64]. In humans, GIT solutes of about MW 500–600 preferentially enter EHC [64]. This MW range includes many flavonoid (i.e., C_6_-C_3_-C_6_) glycosides and glucuronide conjugates. Lower MW phenolic acids (i.e., C_6_-C_n_) and esters, including chlorogenic, ferulic, and caffeic acids, appear to be much less readily taken up into EHC based on studies using surgically cannulated rats [65]. Although many factors influence biliary uptake [64] differences in EHC uptake significantly distinguish the pharmacokinetics of the C_6_-C_3_-C_6_ and C_6_-C_n_ products arising from anthocyanins. 

### 5.2. Flavonoids in EHC

Large, structure-specific effects were found in the secretions of various flavonoids into bile, using surgical cannulation with perfusion in rats [66]. Among six classes of flavonoids, secretion into bile ranged from 1% for the flavan-3-ol catechin, to 32% for the isoflavone genistein. Plasma content for genistein was 1.34 μM which was five times higher than for catechin (0.24 μM) [66]. The high bioavailability of genistein was due to high uptake into bile and EHC [67]. Aspects of enteric and enterohepatic pathways for various flavonoids have been reported [45,63,68] and reviewed [69]. 

### 5.3. Anthocyanins and EHC

Varied evidence suggests that anthocyanins can be taken up into EHC. Like other flavonoid classes, anthocyanins, anthocyanidins and their phase 2 conjugates are in the approximate MW range of 500–600; that is, they are amenable to uptake into EHC by humans [64]. Anthocyanin products with the C_6_-C_3_-C_6_ structure are strongly associated with bile phospholipids in urine. Indeed anthocyanin and anthocyanidin metabolites co-purified with highly amphipathic bile phospholipids in urine, although > 90% of the dry matter was removed during solid phase extraction [61].

During EHC, the sporadic release of bile gives rise to delayed clearance and even multiple peaks in excretion, which has been reported for delphinidin and petunidin glucoside and their glucuronides, after grape juice intake [70]. By using a Ussing chamber and other sections of the mouse GIT, anthocyanin uptake was shown to be highest in the jejunum [54] which is a major site of EHC uptake. Methylated and glucuronidated C3g conjugates appeared in bile soon after C3g was introduced into the small intestine [23]. 

Intestinal absorption of pelargonidin rutinoside was increased from 0.5% to 18% when biliary flow was surgically interrupted, suggesting a significant capacity for uptake by pelargonidin rutinoside into EHC [71]. Six minutes after grape anthocyanins were administered to the gastric cavity, portal and systemic blood flows were 0.65 and 0.234 μM respectively [72], showing that a significant fraction of portal flow was available for biliary circulation.

## 6. Structural Specificity in Anthocyanin’s Behavior in Vivo

An often-asked question regarding anthocyanin’s health benefits is whether the six major anthocyanidins differ in their ADME, and how this may relate to their in vivo bioactivity. Investigating the xenobiotic methylation and hydroxylation of anthocyanins is challenging based on MS/MS because anthocyanidins (i.e., cyanidin, delphinidin, malvidin, pelargonidin, peonidin, and petunidin) are themselves differentiated from each other by their arrangement of methyl and hydroxyl groups on the B-ring. For example, although peonidin occurs in plants, peonidin (i.e., methyl cyanidin) also arises in animals from phase 2 methylation of cyanidin. Similarly, the removal of functional groups will interconvert anthocyanidins; for example, the loss of a hydroxyl group from the B-ring of cyanidin gives rise to pelargonidin [53,56]. Methylation and glucuronidation occurs at hydroxyl groups, which are abundant in anthocyanins and other flavonoids (Figure 1). Therefore, positional isomers of anthocyanin and anthocyanidin conjugates are predicted and indeed detected [21,41,53,55,56,59,73]. Greater complexity in the pool of C_6_-C_3_-C_6_ metabolites can be expected because of EHC and prolonged exposure to enteric and hepatic phase 2 metabolism. 

### 6.1. Cyanidin Metabolism and Products

Cyanidin is the most widely distributed of the six major plant anthocyanidins and also the best-studied with respect to food and health. Isotopically-labeled C3g was used to definitively examine the absorption and metabolism of ^13^C C3g in humans [18] and ^14^C C3g in mice [74]. In the human study [18] at 48 h after intake, 44% of the ^13^C label had been excreted in urine (5.4%), breath (6.9%), and feces (32.1%). The exhalation of ^13^CO_2_ derived from anthocyanin catabolism, which demonstrated complete catabolism of C3g, continued throughout the 48 h study [18]. Also striking was that more than 50% of the ^13^C label was still in the body at 48 h. The authors attributed slow clearance of ^13^C to EHC, and/or prolonged colonic metabolism and catabolite absorption [18]. Other factors may be interaction with intestinal mucin, protein, food materials, or other matrix components [22]. 

In the mouse study that administered orally, a single dose of ^14^C C3g, almost 90% of the ^14^C label was in the adipose tissue and gastrointestinal tissue and contents, 3 h later. After 24 h, about 50% of the ^14^C was accounted for in feces, and 3.3% in urine [74]. These isotope studies suggest that anthocyanin clearance may be slower in humans than in mice. Whereas the ratio of ^13^C labelled C_6_-C_n_ products and ^13^C-C3g was 42 in humans [18], this ratio was only 6 in mice for ^14^C [74], suggesting a longer period for catabolism in the human GIT. Very large variation in ^13^C pharmacokinetics was noted in the human study. The recovery percentage of ^13^C among eight human subjects ranged from 15% to 99%. Additionally, the maximum plasma concentration of ^13^C ranged from 10 to 2000 nM and T_max_ ranged from 2 to 20 h [18].

The ADME of C3g has been examined using various intervention models and with progressively more advanced approaches to chemical and data analysis. Notwithstanding the diversity of approaches, studies concur that methylation and glucuronidation are major routes of C3g conjugation in vivo. Among three human studies, which tracked C_6_-C_3_-C_6_ metabolites in urine after a single dose of either pure C3g [18], or after C3g-rich blackberries [75], or after boysenberries which contain cyanidin mono-glucosides, di-glucosides, and two cyanidin rutinosides [53], the metabolites detected in all these studies included methyl and glucuronide conjugates of C3g, methyl C3g (i.e., peonidin-3-glucoside, Peo3g), cyanidin, and peonidin [18,53,75]. In one case, excretion of C_6_-C_3_-C_6_ products in urine was still underway after 24 h at levels many times greater than C3g [75].

Also detected in these studies were the unconjugated simple aglycones, cyanidin and peonidin [18,53,75]. Aglycone flavonoids are much better substrates than glycosides as substrates for phase 2 conjugating enzyme [45]; however, anthocyanidins (i.e., aglycones) are very unstable in aqueous conditions. The conditions in vivo that stabilize anthocyanidins are unknown. And it is likely that the fate of anthocyanidins would depend on whether phase 2 conjugation could occur, instead of irreversible C-ring fission with loss of the flavonoid structure. 

A substantial amount of C3g metabolism appears to occur in the liver. When C3g was administered to the stomachs of rats, both C3g and Peo3g were found in bile, whereas only C3g was found in plasma (35). When C3g was administered orally to rats, methylated and glucuronidated cyanidin was detected in plasma [73], but when C3g was given by IV, two methylated isomers of C3g, but no glucuronidated forms, were detected [73].

When C3g was administered by IV, methylated C3g in plasma rose and fell quickly which reflected either absorption (into tissues) and/or decomposition from circulation [76]. Other pathways of xenobiotic metabolism were apparent because small amounts of delphinidin, petunidin, malvidin, and pelargonidin glycoside were also detected in plasma, reflecting C3g methylation, hydroxylation, and dehydroxylation reactions [76]. With respect to the area under the curve for plasma, the ranking was C3g > Mal3g > Peo3g > pelargonidin-3-glucoside [76]. Similar results were obtained in another study where, after C3g injection, trace amounts of the 3-glycosides of malvidin > delphinidin > petunidin, occurred transiently in the plasma and kidneys which reflected xenobiotic methylation and hydroxylation capacity [77].

### 6.2. Malvidin Metabolism and Products

Malvidin is the largest, most methylated, and most hydrophobic of the six common food anthocyanidins, and for this reason has a somewhat distinctive ADME [78]. Among 13 studies that qualified for inclusion in a systematic review on anthocyanins in animal tissues, the highest concentration reported was that of Mal3g which was found in brain tissue (4.43 pmol/g) of young swine that were fed bilberry extract at 82.5 mg/kg/day for 3 weeks [79]. In another study with swine, three malvidin glycosides (galactoside, glucoside, and arabinoside) were the most abundant among 12 anthocyanins detected in two brain regions, eyes, and livers of blueberry-fed pigs [80]. Mal3g content contributed at least 50% to the total anthocyanins detected in these tissues [80]. Mal3g and malvidin-3-galactoside were the most abundant of ten anthocyanins detected in brain tissue [81]. Mal3g and malvidin-3-galactoside were predominant in the plasma and tissues of mice after both a single dose and long-term feeding of bilberry anthocyanins although malvidin glycosides were only about 15% of the total bilberry anthocyanins [82]. This result illustrates the capacity for phase 2 methylation of anthocyanins in vivo in mice.

In a long-term study with humans (*n* = 17) consuming blueberry juice, malvidin glycosides (including the 3-glucoside, galactoside, and arabinoside) were three of only four anthocyanins that were statistically grouped among the 55 most abundant urinary anthocyanin metabolites [56]. The other abundant C_6_-C_3_-C_6_ metabolites were mostly deglycosylated forms, including methyl and glucuronide conjugates of all six common anthocyanidins [56]. Together, these pig and rodent studies examining tissues and fluids suggest that malvidin glycosides may be resistant to metabolism due to their bulkiness and degree of methylation [83]. The enzymatic capacity for methylation of petunidin glucoside to form Mal3g has been documented [84]

### 6.3. Pelargonidin Metabolism and Products

During anthocyanin breakdown in vivo, human and microbial-catalyzed demethylation and dehydroxylation of more highly substituted anthocyanins and anthocyanidins gives rise to pelargonidin. The removal of functional groups to yield pelargonidin glycoside and pelargonidin helps to explain the high apparent recovery of pelargonidin-based metabolites [21,85,86,87]. Pelargonidin glucuronide was detected in urine and attributed to cyanidin dehydroxylation in a boysenberry study where four different cyanidin glycosides were fed to humans [53]. Pelargonidin glucuronides could also arise from the catabolism of more highly substituted anthocyanidin glucuronides. Pelargonidin-3-glucoside was detected when Cy3g was administered by IV [76].

Blueberries contain only five of the six types of anthocyanidins; they do not contain pelargonidin glycosides [88]. During long term blueberry juice intake, pelargonidin-based metabolites, although absent from blueberry juice, were abundant in urine [56] when detected using HPLC-MS/MS. It is interesting to consider whether pelargonidin glycosides could serve as a flavonoid-based biomarker of anthocyanin intake. Indeed, pelargonidin was determined to be the most membrane soluble among a selection of anthocyanidin glycoside and aglycones [28].

## 7. Anthocyanin Retention in Membranes and Tissue

### 7.1. Anthocyanins in Tissues

In a recent review, two hundred seventy nine publications on the topic of anthocyanin tissue bioavailability were identified, of which thirteen met the review’s inclusion criteria [78]. Of the thirteen, five studies examined anthocyanin tissue bioavailability after a single dose [55,77,89,90,91]. Four studies examined a single IV administration of anthocyanins [55,76,77,90]. Six studies examined anthocyanin in tissues after long-term feeding in rodents and pigs [36,79,81,82,89,92]. Parent anthocyanins and their phase 2 metabolites have been detected in many tissues where they have been sought, with exceptions [37,82,91]. Among the thirteen studies reviewed in detail, anthocyanin content was greatest in mice kidneys (2.17 × 10^5^ pmol/g), liver (1.73 × 10^5^ pmol/g), heart (3.6 × 10^3^ pmol/g), and lungs (1.16 × 10^5^ pmol/g), and in pig brain (6.08 × 10^3^ pmol/g) [78]. 

Anthocyanin appears to have a high affinity for tissues. In a blueberry study, where control pigs consumed a basal (0% blueberry) diet that contained anthocyanins from grain equivalent to 0.0002% of the blueberry powder, measurable amounts of parent anthocyanins were detected in pig tissues [80]. Although it is not possible to analyze human tissue for anthocyanin, there is no obvious reason why anthocyanins would not also be in human tissue. 

Studies that were not included in the review by Sandoval-Ramirez also report anthocyanin in tissues of rodents following a single dose of berries [93,94] and in pigs during long-term anthocyanin feeding [80]. In one rat-feeding study, parent anthocyanins and some phase 2 methylated and glucuronidated forms were detected in GIT, liver, kidney, and brain tissues [93]. Among GIT tissues, anthocyanins were 10-times more abundant in the jejunum, which is a major site of EHC uptake, compared to other gastric tissues [93]. 

### 7.2. Anthocyanin Beyond the Blood Brain Barrier

Among the thirteen studies reviewed in detail [78] seven studies attempted to detect anthocyanin in brain tissues after either a single anthocyanin dose [76,82,90] or after long term anthocyanin feeding [37,79,81]. In two single-dose studies, anthocyanin was detected in other tissues but not the brain [82,95]. In a long term feeding study [37], anthocyanins were not detected in plasma or tissues, which may be attributed to the high anthocyanin dose fed (8% blueberries), as discussed earlier.

Anthocyanins were detected in brain tissue within 10 min after grape anthocyanins were incubated in the gastric cavity. For some of the grape anthocyanins, levels in brain tissue were comparable to levels found in plasma [94]. When anaesthetized rats received C3g by IV injection, C3g was detected in the brain tissue within 15 sec and found at a concentration comparable to that in serum [76]. Indeed, anthocyanin content in the brain was found to correlate with plasma levels over a wide range [76]. When anthocyanin was administered by IV, fewer anthocyanin metabolites were found in brain tissue compared to plasma, suggesting carrier-mediated transport across the blood brain barrier [76]. 

In a study where a bilberry-enriched diet was fed to young swine for 3 weeks, dose-dependent levels of malvidin-, delphinidin- and cyanidin-galactoside and petunidin, Peo3g, and C3g were reported in various brain regions [79]. In another study where pigs were fed 2% for blueberries ad libitum for 8 weeks, phase 2 metabolites of anthocyanins were detected in the brain, including glucuronides of cyanidin, malvidin, peonidin, delphinidin, and petunidin. These were identified in the cortex, cerebellum, and the midbrain, plus in the diencephalon in combination [81]. 

Anthocyanin was detected in the whole eye of pigs that were fed 0%, 1%, 2%, and 4% blueberry powder, in a manner that appeared to be dose dependent [96]. Following intraperitoneal administration of anthocyanins to rats, they were detected in the whole eye and in some ocular tissues at a level higher than in plasma [97]. When anthocyanins were administered intraperitoneally to rabbits, they were differentially distributed among several ocular tissues, and were particularly rich in the connective tissue of the sclera and cornea [97]. 

## 8. How Anthocyanin-Derived C_6_-C_3_-C_6_ Products May Work in Vivo

Parent anthocyanins can survive transit through the GIT and be excreted intact in feces, which is evident by the purple anthocyanin pigmentation seen in the feces of birds and other animals that consume large amounts of berries. 

### 8.1. The Pool of Anthocyanin Products in the Body

Products arising from anthocyanin metabolism and catabolism either possess or not, the diphenyl propanoid backbone characteristic of flavonoids. Many papers suggest that non-flavonoid breakdown products (i.e., catabolites) of anthocyanins, i.e., C_6_-C_n_, phenolic acids, and aldehydes, are responsible for health benefits [98,99,100,101]. To determine the relative contributions of C_6_-C_3_-C_6_ and C_6_-C_n_ metabolites to anthocyanin health benefits, it is important to note that some C_6_-C_n_ phenolic products are present in other fruits and vegetables (e.g., syringic, vanillic, and gallic acids) [102] that are devoid of anthocyanins. Indeed, catabolites of anthocyanins contribute to an already very large pool of low MW phenolic moieties in the GIT. Further evidence for a special role for anthocyanins in health is that abundant phenolic moieties do not explain the in vitro effects of parent anthocyanins. Additionally, C_6_-C_n_ phenolics cannot explain the growing epidemiological evidence that specifically associates moderate anthocyanin intake with disease risk reduction and improved health outcomes [6,7,8,14,15,16]. 

### 8.2. Slow Clearance of C_6_-C_3_-C_6_ Metabolites from Human Urine

In a long-term blueberry juice intake study, multiple positional isomers of methylated and glucuronidated anthocyanin and anthocyanidins were detected in human urine, for a total of 45 major C_6_-C_3_-C_6_ conjugates. These products were detected using LC-MS/MS analysis of 18 parent and 43 predicted metabolites for all six anthocyanidins [35,56]. The feeding trial also demonstrated the protracted release of C_6_-C_3_-C_6_ metabolites, even after no anthocyanin intake for 5 days [61]. The continued release of C_6_-C_3_-C_6_ metabolites was attributed to their release from EHC and tissue reservoirs. These findings are supported by a long-term, placebo-controlled blueberry study in humans showing even more protracted release of C_6_-C_3_-C_6_ metabolites in the urine of the placebo group, while cognitive improvements were associated with parent anthocyanins in the urine of the blueberry group (Krikorian et al. in press). 

### 8.3. How C_6_-C_3_-C_6_ Products from Anthocyanins May Work in Vivo

It is plausible that anthocyanin-derived products could be associated with the biomatrix and escape detection using conventional analytical approaches. This aspect would support a role of anthocyanin products in antioxidant protection. Abundant anthocyanins in the GIT, both at the membrane surface and in intestinal contents, can act as antioxidants against reactive oxygen species in the GIT, especially in the colon where the levels of Vitamin C and carotenoids are limited, but flavonoids are present [103]. Antioxidant protection may also be provided to tissues where anthocyanidin-derived C_6_-C_3_-C_6_ products are localized. Molecular modelling studies have shown that some flavonoids, including quercetin, can be positioned at the membrane-aqueous interface and contribute to antioxidant defense in the membrane by shuttling protons from the aqueous phase [104,105]. 

Systemic delivery of anthocyanins and anthocyanidins throughout the body was hypothesized to occur via a ‘glucuronide conveyor’ [27]. According to the hypothesis, polar glucuronides reach peripheral tissues via systemic circulation. Then glucuronides are deconjugated by peripheral glucuronidase activity, releasing anthocyanidins or anthocyanins of reduced polarity which are available for association with the biomatrix and for tissue uptake. Notably, peripheral glucuronidase activity can be affected by stresses such as inflammation [27]. Such a conveyor could provide a means to widely distribute anthocyanin-derived C_6_-C_3_-C_6_ products throughout the body.

The possible broad deposition of anthocyanins in body fluids and tissues raises an interesting issue with respect to rodent versus human research models. Whereas lab animals will have had no previous exposure to anthocyanins, humans will have had life-long dietary exposure to them. This factor could affect the capacity to absorb and respond to anthocyanins by rodents in comparison to humans.

## 9. Methods to Study Anthocyanin Bioavailability

The review on anthocyanin deposition in tissues [78] stated that the greatest limitation in comparing research findings was the heterogeneity in the research methodologies, which is indeed valid for all aspects of anthocyanin ADME research. Notably, analytical methods for the ex vivo analysis of anthocyanin in biomatrices continue to improve. LC-MS/MS technologies for sensitive and accurate mass identification have advanced substantially and are well suited for tracking a single drug. 

The difficulty with anthocyanin bioavailability research is first, the large number of parent anthocyanins consumed in plant foods, and second the large suite of flavonoid and non-flavonoid products, including glycosidic and non-glycosidic conjugates emanating from each parent anthocyanin over time and sites within the body. Analytical methods must, therefore, focus on tracking the largest number of predicted moieties possible, and then apply data analytics and modelling to build a more inclusive, rather than reductive model to understand the behavior of anthocyanins in vivo and how they support health. 

Anthocyanin metabolism in vivo affects molecular size and polarity and, as a result, alters metabolite solubility among biomatrices (Table 1). This aspect probably contributes to inconsistencies among reports, including approaches to sample handling and analysis. Targeted sample clean-up [60,89] and optimized LC-MS conditions in positive ion mode, plus data mining and modeling are key requirements in investigating the complex ADME of anthocyanins. Pure analytical standards are mostly not commercially available for use in the identification of C_6_-C_3_-C_6_ based conjugates of anthocyanins and anthocyanidins and therefore, this potentially important group of early anthocyanin metabolites has not been well-studied. Notably, there are no published descriptions of artifacts related to flavonoid conjugate detection using LC-MS/MS. 

## 10. Conclusions

Site-specific conditions contribute to a variety of physical and metabolic events that affect the structure and properties of anthocyanins in vivo (Table 1). As a result, the ADME of anthocyanins involves a diverse pool of both C_6_-C_3_-C_6_ and C_6_-C_n_ metabolites which appear to collectively benefit health in a variety of ways. To date, there is no unambiguous evidence pointing to the bioactivity of any specific anthocyanin product(s) in vivo. Indeed, with consideration of the ecological role of anthocyanins as foods, they do not have the potency nor the behavior of drugs. Instead of acting as single agents, with limited in vivo actions, a multitude of products and actions are derived from anthocyanins in vivo. 

Research perspectives should aim to not be reductionist in nature. While anthocyanin bioavailability research has historically been focused on the low MW phenolic catabolites of anthocyanins, the distinct and diverse chemical properties of various C_6_-C_3_-C_6_, anthocyanin-derived products warrant more attention. These metabolites possess the distinctive flavonoid structure, which is associated with bioactivity. 

Large inter-individual variation is documented in phenotypic factors [36,50] and pharmacokinetics [18] for anthocyanin metabolism in humans. This is mostly due to human, and not microbial metabolism, and is an obstacle in developing generalizable knowledge that can guide future research. 

## Figures and Tables

**Table 1:** Sites of occurrence of anthocyanins and their C_6_-C_3_-C_6_ products in vivo, and the events and effects associated with them.

Site	Event	Effect	Reference
Mouth	Deglycosylation Association with saliva proteins	Polarity decrease; membrane solubility increase Decline in free parent anthocyanin	[22,47]
Stomach	Uptake on bilitranslocase Stabilization by acidic pH	Rapid absorption and distribution of parent anthocyanins	[44,77]
Small intestine	Association with food matrix Association with intestinal mucin High concentration in small intestine Enteric transport and phase 2 metabolism	Increased stability Delayed, impeded absorption Antioxidant effect in small intestine Distribution and formation of phase 2 conjugates	[22,27,103]
Liver	Hepatic phase 2 metabolism	Formation of phase 2 conjugates	[106]
Large intestine	Survival in colon	Darkening and purple coloration of feces (e.g., birds and bears) Antioxidant protection in colon	[103]
Bile	Dissolution of anthocyanins and their metabolites in amphipathic bile	Capacity for enterohepatic circulation due to anthocyanin properties Complex pool of anthocyanin isomers	[56]
Plasma	Association with serum albumin Preferred protein association with anthocyanin glycosides	Increased anthocyanin stability Depot for protein-associated anthocyanin	[32]
Tissues	Association with tissues	Long-term retention and possible protection of membranes and other structures	[78,104]
Liposomes and micelles	Reduced water activity	Increased anthocyanin stability Differential distribution and mobility in membranes	[25,26]

## Abbreviations

ADME	absorption, digestion, metabolism and excretion
C3g	cyanidin-3-glucoside
EHC	enterohepatic circulation
GIT	gastrointestinal tract
Mal3g	malvidin-3-glucoside
MEKC	micellar electrokinetic capacity
Peo3g	peonidin-3-glucoside
